# Prussian Blue and Carbon-Dot Hybrids for Enhanced Electrochromic Performance

**DOI:** 10.3390/ma14123166

**Published:** 2021-06-09

**Authors:** Jia Chu, Yaping Cheng, Xue Li, Fan Yang, Shanxin Xiong, Zhao Zhang

**Affiliations:** 1 Department of Chemistry, Zhejiang University, Hangzhou 310027, China; 2 College of Chemistry and Chemical Engineering, Xi’an University of Science and Technology, Xi’an 710054, China; 18991634831@163.com (Y.C.); lixuexust@163.com (X.L.); yfwy123@126.com (F.Y.); 3 Zhejiang Yuxi Corrosion Control Co., Ltd., Ningbo 315700, China

**Keywords:** Prussian blue, carbon dot, hydrothermal, electrochromic

## Abstract

In this study, Prussian blue@Carbon-dot (PB@C-dot) hybrids have been developed by one-step hydrothermal method. The incorporation of C-dots into Prussian blue thin film as a way of improving its electrochromic performance was investigated. The structure of the PB@C-dot hybrid was characterized through X-ray diffraction, Raman spectroscopy and scanning electron microscopy. The electrochromic properties showed that incorporation of 10 mL C-dots into the film showed higher optical contrast of 1.6 and superior coloration/bleaching response of 10 and 3 s. It is proposed that the C-dots component used in the construction of the PB@C-dot hybrid plays a key role to achieve superior electrochromic performance.

## 1. Introduction

Electrochromic materials have attracted much attention because of their potential applications in antiglare mirrors, smart windows and sunglasses [1,2,3]. Widely researched electrochromic materials include organic conductive polymers, inorganic metal oxides and Prussian blue [4]. These materials are usually used in form of flexibility electrochromic devices (ECDs), thin film devices, electrochromic windows and smart windows and so on. Among them, Prussian blue (PB) is an extensively studied electrochromic material with low cost, ease of synthesis and stable electrochemical redox behaviors [5,6]. However, PB exhibits poor electrochemical conductivity, which limits the development and practical uses of this material in the field of electrochromic devices (ECDs) [7]. Therefore, it has become desirable to effectively improve the conductivity of PB film and avoid the leakage form the electrode surface to meet the requirements of their applications in ECDs. To address the limitations and achieve improved electrochemical properties, one of the most effective ways is to provide synergistic effects in the composition form, such as carbon-based nanocomposites. Carbonaceous materials such as carbon nanotubes, graphene and reduced graphene oxide are often added to the electrochromic layer to improve the conductivity and stability [8]. These hybrids showed enhanced electrochromic performances due to the incorporation of carbonaceous material improves the reversibility of electron transport and ionic motion [9,10,11,12]. A drawback of using graphene and carbon nanotubes as an incorporation material is that the composites often suffer from high contact resistance, phase separation and difficult synthesis. However, there are works on alternative sources methods of obtaining them [13,14,15]. The key point is to find an optimal way with facile synthesis route and unique electronic properties [16]. Even so, nanocomposites with high contrast ratio, prolonged cycling stability and rapid switching kinetic for PB composites are still seldom achieved.

Supported layers of PB can be grown by several synthesis methods, including electrochemical deposition [17], spin-coating [18] and hydrothermal [19,20]. Among all synthesis methods, hydrothermal growth has led to effective composition for preparation of PB and their composites. Recently, C-dots are considered as a new class of carbon nanomaterial and used in many research fields [21]. For example, Shen et al. [22] studied a photoelectrochromic device (PECD) consisted of carbon quantum dots (CQDs) sensitized photoanode, poly(3,4-(2,2-dimethylpropylenedioxy)thiophene) (PProDOT-Me_2_) electrochromic film, Br^−^/Br^3−^ with Li^+^ as electrolyte was fabricated, and it shows advantages of high transparency in a bleached state when compared with conventional PECD. Tina et al. [23] reports the synthesis of CQDs from chitin nanofibers (CNFs) as a new carbon source via microwave-assisted hydrothermal route within three minutes. The results shown that CQDs in the presence of Cu^2+^ ions can be successfully applied to the determination of DPA in human blood serum, urine and pharmaceutical samples. Costa et al. [24] described two simple and low-cost routes for the acquisition of CQD materials based on peat. The main characteristic of CQDs produced from peat are their photoluminescence, which has the potential for use in optoelectronic devices in organic electronics or even in sensors for detecting toxic agricultural inputs in the water of rivers and lakes. Janus et al. [25] proposed a new, ecofriendly bottom-up synthesis approach for intelligent, surface-modified nanodots preparation using bioproducts as a raw material. The obtained results show that proposed carbon nanodots can be applied in medicine and pharmacy as elements of advanced sensors, optical fibers or for cell bioimaging and biochemical processes study.

**Figure 1:**
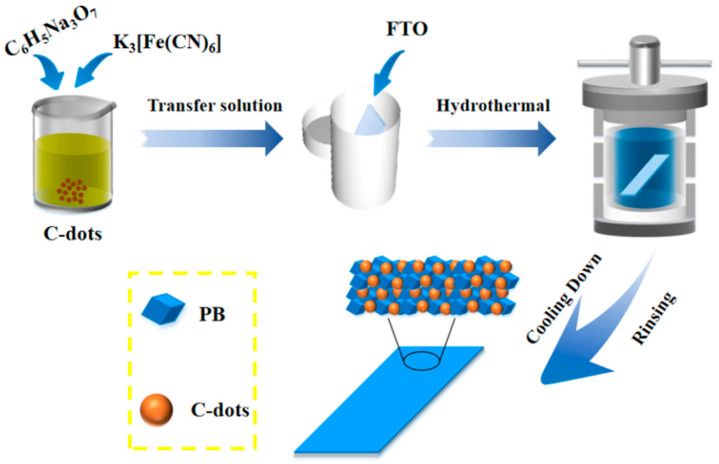
Schematic for fabrication of PB@C-dot composite film.

In comparison to other materials, C-dots possess a unique nanostructure with exceptional electronic, optical, thermal and ultrahigh surface areas. In this regard, C-dots are considered to be an ideal candidate for overcoming most of the aforesaid limitations about Prussian blue-modified electrodes. To the best of our knowledge, little has been written on the use of C-dots as components for electrochromic material [20]. In this research, the incorporation of C-dots into PB thin films is investigated as a means to improve the electrochemical property of this electrode. We first obtained C-dots via a reported method [26]. C-dots composited with PB grown on FTO glass were fabricated through a hydrothermal reaction. Further, the electrochromic properties of these PB@C-dot composites were measured. The fabrication processes are illustrated in Figure 1. The results suggest that the obtained PB@C-dots hybrid is a promising candidate in terms of electrochromic application.

## 2. Experiment

### 2.1. Synthesis of PB@C-Dot Hybrid

All the chemicals are of analytical grade and used without any further purification. Firstly, the C-dots were prepared according to the reported method [26]. Briefly, 3.153 g of citric acid monohydrate (CA) and 1 mL of Ethylenediamine (EDA) were dissolved in 30 mL of deionized water. After stirring for 20 min, the mixture was transferred into a Teflon-lined stainless-steel autoclave and then kept at 180 °C for 24 h. The autoclave was cooled down to room temperature naturally. Then, the C-dots were separated by being dialyzed in a dialysis bag (retained molecular weight: 1000 Da) with deionized water for 2 days. C-dots solution was then collected and used thereof. The PB@C-dot hybrid was grown on FTO glass substrate by hydrothermal method. In a typical procedure, 0.66 g potassium ferricyanide (K_3_[Fe(CN)_6_]) and 0.25 g trisodium citrate (C_6_H_5_Na_3_O_7_) were dissolved in the mixed solution of C-dots and distilled water; among them, the six different volume ratios of C-dots/H_2_O were chosen, i.e., 0/60, 2/58, 5/55, 10/50, 20/40 and 30/30, respectively. Afterward, 0.4 mL 36% hydrochloric acid (HCl) was dropped into the solution. After stirring for 20 min, the resulting solution was transferred into a 50 mL Teflon-lined stainless-steel autoclave containing a FTO substrate (1 × 5 cm^2^ in size) with the conducting side facing down. Then, the sealed autoclave was maintained at 120 °C for 1 h. After cooling naturally to room temperature, the product was washed with distilled water and dried at 60 °C in a vacuum oven. The final products were denoted as PB@C-dot-0, PB@C-dot-2, PB@C-dot-5, PB@C-dot-10, PB@C-dot-20 and PB@C-dot-30, respectively.

### 2.2. Characterization

The crystal structure and surface morphology of the PB@C-dot films were investigated by X-ray diffraction (XRD, Rigaku DMAX 2400) with Cu Kα radiation and scanning electron microscopy (SEM, Hitachi S-4800, Japan). Raman spectra were recorded on an Invia Renishaw spectrometer using an excitation light wavelength of 532 nm. Electrochromic properties of these films were measured via UV-Vis spectrophotometer (Shimadzu UV-2550, Japan), by applying constant potentials and square-wave potentials using an Autolab PGSTAT302N workstation. Cyclic voltammetry (CV) and electrochemical impedance spectroscopy (EIS) measurements were conducted on a CHI660E electrochemical workstation using a three-electrode system with 1 M KCl as the electrolyte, PB@C-dot film as the working electrode, saturated calomel electrode (SCE) as the reference electrode and Pt sheet as the counter electrode, respectively.

## 3. Results and Discussion

**Figure 2:**
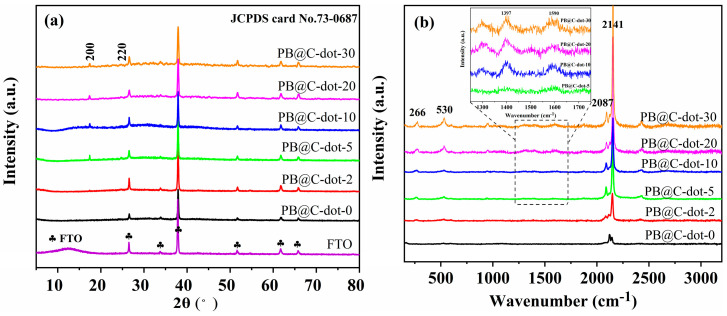
(**a**) XRD patterns. (**b**) Raman spectra of the as-synthesized PB@C-dot films.

XRD was applied to characterize the crystal phase of the PB@C-dot films. As shown in Figure 2a, the film exhibits two diffraction peaks at 2θ values of 17.4 and 24.6° (after subtracting the diffraction peaks of FTO glass), which can be indexed as (200) and (220) crystal planes of PB (JCPDS No. 73-0687) [27]. All diffraction peaks suggest high crystallinity and no significant impurities in the as-synthesized PB@C-dot composites. The film mixing with C-dots exhibits more intensive PB crystal peaks than that without C-dots. Notably, due to the relatively small amount of the C-dots, the peaks related to the C-dots were barely observed in the patterns of PB@C-dot films. The existence of C-dots phase can also be characterized by Raman spectra. Raman spectra (Figure 2b) show that the strong bands located at 2087 cm^−1^ and 2141 cm^−1^ correspond to the stretching vibration of the C≡N bond from the cyanide ligands in PB. The bands at 266 cm^−1^ and 530 cm^−1^ are assigned to Fe-C, Fe-CN and Fe-N modes [9,28]. Meanwhile, it can be observed (inset) that the PB@C-dot exhibits the characteristic peaks of C-dots at 1397 cm^−1^ (D-band) and 1590 cm^−1^ (G-band), suggesting that the C-dots were involved in the PB growth [29].

**Figure 3:**
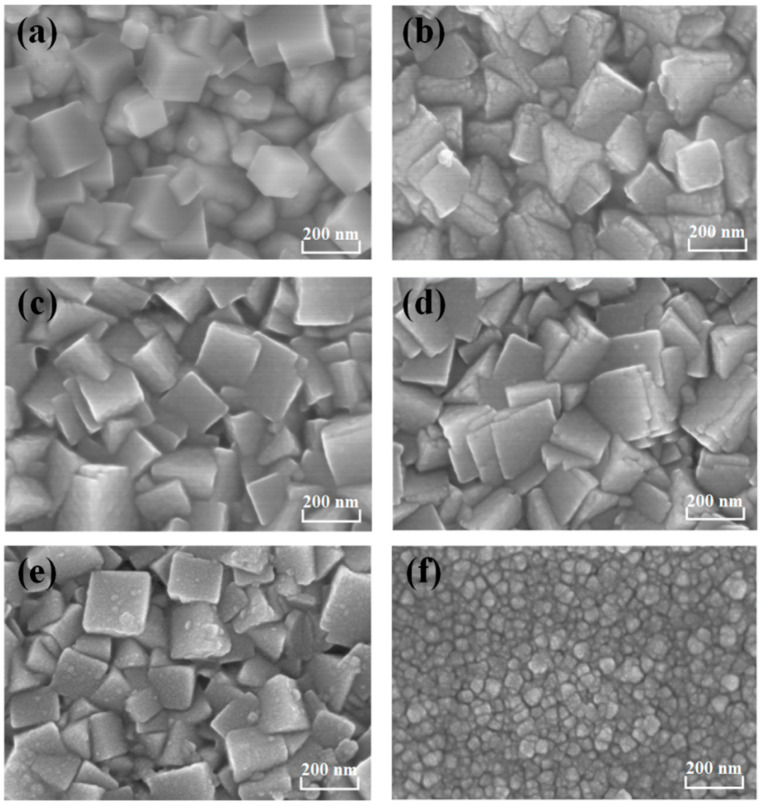
SEM images of (**a**) PB@C-dot-0, (**b**) PB@C-dot-2, (**c**) PB@C-dot-5, (**d**) PB@C-dot-10, (**e**) PB@C-dot-20 and (**f**) PB@C-dot-30.

**Figure 4:**
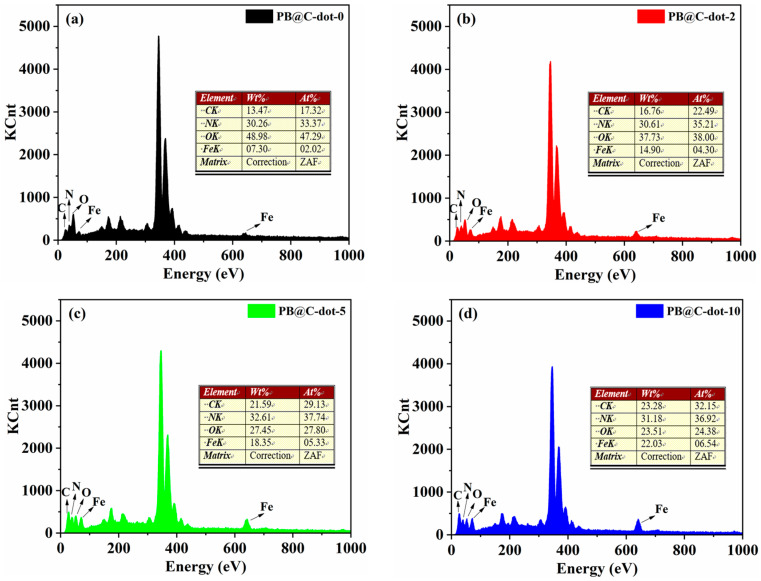

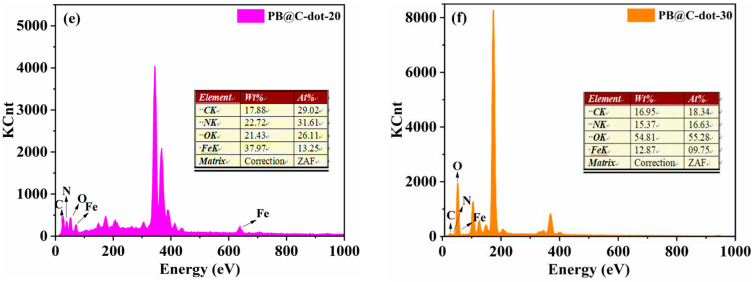
EDAX results of the (**a**) PB@C-dot-0, (**b**) PB@C-dot-2, (**c**) PB@C-dot-5, (**d**) PB@C-dot-10, (**e**) PB@C-dot-20 and (**f**) PB@C-dot-30.

The surface morphology of the PB@C-dot films were investigated by SEM, as presented in Figure 3. Before the C-dots were added, the entire surface of the FTO substrate was covered with PB nanocubes, forming a relatively compact morphology with an average diameter of 100–200 nm (Figure 3a). The morphology of the PB@C-dot layers hydrothermally grown on FTO glass can be composed of two different morphologies. For the hybrid containing 2 mL C-dots, the nanocubes of PB@C-dot-2 film with slightly collapsed angles are stacked, as shown in Figure 3b. In the case of PB@C-dot-5, the nanocubes are tightly embedded together (Figure 3c). When more C-dots were added, quite different morphology was observed for the hybrid containing 10 mL C-dots, which showed obvious coalescence with crystals in a particle-like morphology (Figure 3d). These different characterization results demonstrate the large effect of the absence or presence of C-dots on the surface morphology of the composite layer. We proposed that the C-dots may have interconnected with the PB nanocubes during the hydrothermal process. The smaller PB@C-dot-30 particles were coated on the surface of FTO glass. The smoothness of the PB@C-dot-30 film can be attributed to the doping of C-dots, which induces the secondary nucleation and decreases the particle size of the crystal. The SEM images and EDAX results suggests the formation of a PB@C-dots composite film. They also show the PB@C-dot hybrid films are well-arranged crystalline, which is uniform and dense without holes or cracks. Furthermore, EDAX measurement confirms the presence of C, N, O and Fe element for the PB@C-dot films. The atomic% and weight% of the above elements are presented in Figure 4 (inset Table). The results show that the addition of 2 mL, 5 mL and 10 mL C-dots into the PB film enhance the weight% of C element of the thin film. When the 20 and 30 mL C-dots were added, the C element decreased due, in part, to poor involvement of excessive C-dots during the reaction. The SEM images and EDAX results suggest the formation of a PB@C-dots composite film.

**Figure 5:**
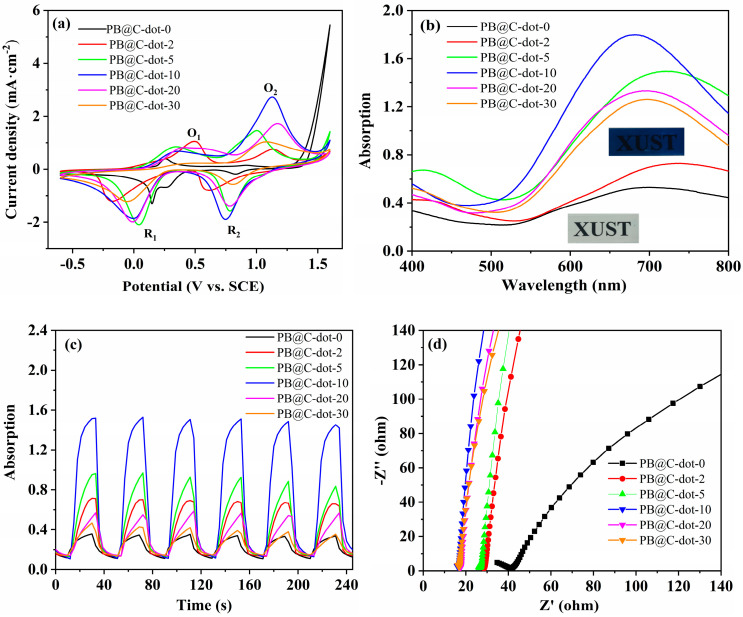
(**a**) CV curves. (**b**) UV-Vis spectra and digital photographs under visible region. (**c**) The switching characteristic at 680 nm and (**d**) EIS spectra of all the PB@C-dot films.

In order to understand the electrochemical performance of PB@C-dot films, the CV curves were estimated in the potential range from −0.6 to 1.6 V at a scan rate of 50 mV s^−1^, as shown in Figure 5a (the CV curves of films at various scan rates are presented in Figure S1). It can be seen that two pairs of distinct redox peak in all the samples, which are ascribed to the oxidation reaction (Fe^2+^→Fe^3+^) and reduction reaction (Fe^3+^→Fe^2+^), respectively [30,31]. Comparatively, the peak area and peak positions of the CV curves in the PB@C-dots exhibit large variations under same scan rate. The PB@C-dot-0 present two pairs of redox peaks: one pair of redox peaks centered at 0.858 and 0.825 V corresponds to the color changes between PB and PG, and another pair of redox peaks centered at 0.267 and 0.145 V corresponds to the color change between PB and PW [32,33]. When the C-dots were added, the positions of oxidation and reduction peaks shifted toward a more oxidation and reduction peak directions. Interestingly, the PB@C-dot-10 film exhibited higher current density and larger enclosed areas than other films, which can be attributed to the C-dots playing a key role in facilitating electron transfer and ionic motion throughout the film [34,35]. However, it is noteworthy that the current density decreased for higher contents of C-dots, implying that C-dots need a proper composition dosage. The cycling stability of the hydrothermally grown PB@C-dot hybrids film was investigated by CV curves, which were recorded in 1 mol/L KCl electrolyte for 100 cycles at room temperature (See Figure S2). The areas enclosed by the CV curves slight decreased after 100 cycles, showing a good cycling stability of PB@C-dot.

Figure 5b shows the UV-Vis absorption spectra of the as-prepared films in the wavelength range of 400–800 nm, with potentials at 2 V. The color of the films changes from transparent (bleached state) to dark blue (colored state), as shown in inset Figure 5b. As observed, the optical contrasts for PB@C-dot-0, PB@C-dot-2, PB@C-dot-5, PB@C-dot-10, PB@C-dot-20 and PB@C-dot-30 are 0.31, 0.52, 1.29, 1.60, 1.12 and 1.06, respectively. With the addition of C-dots from 0 to 10 mL, the optical contrast of the PB@C-dot films increased in turn. Nevertheless, the optical contrast for PB@C-dot-20 and PB@C-dot-30 was found to decrease due to the excessive addition of C-dots; this result was confirmed later in this work by switching characteristic test. The kinetic switching response of the PB@C-dot films was carried out at a wavelength of 680 nm by applying a potential of ±2.2 V for 20 s, as shown in Figure 5c. The coloration (*t_c_*) and bleaching time (*t_b_*) of the PB@C-dot films can be evaluated according to the time to reach 90% of the entire change in absorbance. The results of the study are summarized in Table 1. The *t_c_/t_b_* of electrode containing no C-dots was 15/12; the addition of 5 mL and 10 mL C-dots quickened this to 12/6 and 10/3, respectively. The *t_c_/t_b_* for the electrode containing 30 mL C-dots was 15/10. Notably, PB@C-dot-10 hybrid had a slower coloration time (10 s) compared to its bleaching time (3 s). This result may be explained by a slower interaction of K^+^ ions and electrons, leading to a slower coloring speed. Furthermore, the accumulated K^+^ ions had a higher diffusion ability; therefore, they required the shortest bleaching time [36]. This result indicated that simply by adding an optimum amount of C-dots into a PB film can significantly accelerate the coloration/bleaching time of this electrode, which is consistent with that previously observed in cyclic voltammetry. These improved results for the PB@C-dot films are attributed to the reinforcement of the electrochemical activity of C-dots, which facilitates the insertion or extraction process of K^+^ ions and electrons within the PB@C-dot film. In order to further demonstrate the electrochemical behaviors of the PB@C-dot films, EIS was carried out within the frequency range from 10^−2^ to 10^5^ Hz. As shown in Figure 5d, each Nyquist plots of the films are composed of semicircles in the high frequency region and a sloping line in the low frequency region to represent charge transfer resistance (Rct) and diffusion resistance, respectively [37]. As expected, the resistance values of all films decreased with the incorporation of C-dots. In addition, it is clearly presented that the PB@C-dot-10 exhibited higher slope, indicating higher ion diffusion rate among other films. This result illustrated that the incorporation of C-dots with PB can enhance the electrical conductivity and thus improves its electrochromic performances. Based on the structural characterizations and electrochemical properties, the improved behaviors were mainly due to the enhanced electron transfer via synergistic effect between PB and C-dots.

## 4. Conclusions

This study investigated the effects of the addition of C-dots on the electrochromic performance of PB film. The accelerated optical modulation and fast coloration/bleaching efficiency show that the addition of C-dots into the PB film enhances the electrochromic performance of the electrode, with 10 mL C-dots additions accelerating the coloration/bleaching time of the electrode notably. The excellent electrochromic performance is mainly attributed to the efficient charge transfer ability of C-dots. In this integrated system, the merits of both C-dots and the PB may create synergistic effects and lead to very high electrochromic efficiency and superior optical modulation. Thus, the incorporation of C-dots into the electrochromic materials maybe useful to improve both electrochromic performance and electrochemical behavior.

## Figures and Tables

**Table 1:** The coloration (*t_c_*) and bleaching time (*t_b_*) of all the PB@C-dot films.

Samples	Coloration Time (*t_c_*/s)	Bleaching Time (*t_b_*/s)
PB@C-dot-0	15	12
PB@C-dot-2	14	7
PB@C-dot-5	12	6
PB@C-dot-10	10	3
PB@C-dot-20	14	9
PB@C-dot-30	15	10

## Data Availability

The data presented in this study are available on request from the corresponding author after obtaining permission of authorized person.

## Supplementary Materials

The following are available online at https://www.mdpi.com/article/10.3390/ma14123166/s1, Figure S1: CV curves of (a) PB@C-dot-0, (b) PB@C-dot-2, (c) PB@C-dot-5, (d) PB@C-dot-10, (e) PB@C-dot-20 and (f) PB@C-dot-30 at different scan rates of 10, 20, 50 and 100 mV s^−1^. Figure S2: CV curves of PB@C-dot-10 at 50 mV s^−1^ after different cycles.
