# Supplementary material for: Prussian Blue and Carbon-Dot Hybrids for Enhanced Electrochromic Performance
Source: Materials (Basel). 2021 Jun 9;14(12):3166. doi: 10.3390/ma14123166 (PMC8227488; doi:10.3390/ma14123166)
Supplement: Supplementary file 1 [file materials-14-03166-s001.zip › materials-1222492-supplementary.pdf]

materials

MDPI

Supplementary Material

Prussian Blue and Carbon-dot Hybrids for Enhanced Electrochromic Performance

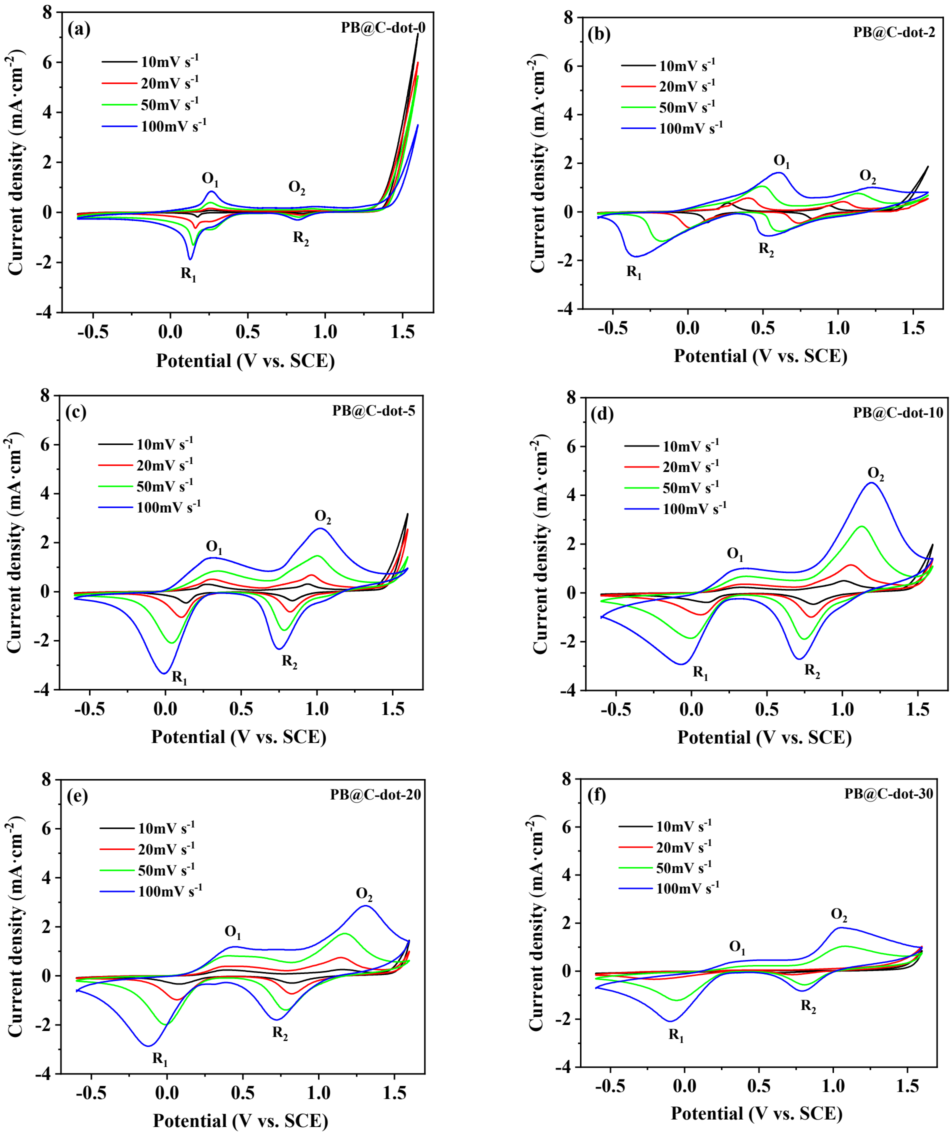

Figure S1. CV curves of (**a**) PB@C-dot-0, (**b**) PB@C-dot-2, (**c**) PB@C-dot-5 (**d**) PB@C-dot-10, (**e**) PB@C-dot-20 and (**f**) PB@C-dot-30 at different scan rates of 10, 20, 50 and 100 mV s^{-1}.

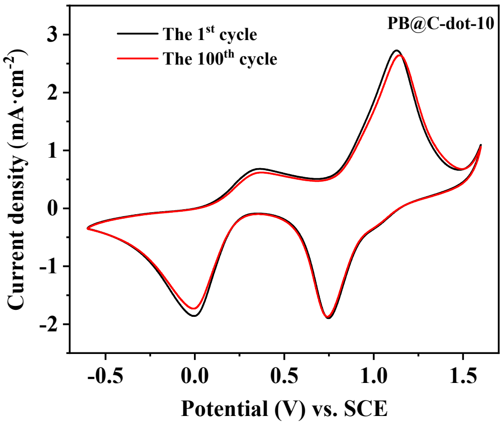

Figure S2. CV curves of PB@C-dot-10 at 50 mV s^{-1} after different cycles.
